# Pulp Mummification

**Published:** 1900-10-15

**Authors:** 


### PULP MUMMIFICATION.

Theodore Soderberg, of Sydney, Australia, presents in the July *Cosmos* a statement of a large number of test cases (220) in which he shows the successful use of the mummifying paste mentioned in the January REGISTER, page 8.

In compiling the table he observed the following rules: (1) Certainty of correct diagnosis; (2) Cavity, tooth structure, etc., to promise at least five years' durability of filling; (3) Certainty of enlargement of chamber to contain sufficient paste; (4) Complete sterilization with peroxide of hydrogen and carbolic acid; (5) Examination of tooth after two years.

Ninety-three of these cases were examined at intervals of from two to three years after treatment, sixty-six cases after three to four years, thirty-nine after four to five years, nineteen after five to six years and three cases after six years. In none of these cases was pericemental or apical trouble traceable to the treatment.

### MODERATION IN EQUIPMENT.

Young man, when you are ambitious "to keep up with the times," just keep cool, and do not get into the fever of belief that the chief and first thing you must do is to explore the catalogues and the depots, and then do your best to outrival your neighbor in the glitter and gorgeousness of mahogany chairs and silver-plated spittoons. One of the best, and, indeed, one of the necessary things you must do, or should do, is to visit the depots and read their advertisements. To-day they are quite as educational, in a practical sense, as the colleges, and afford you free facilities for enlightenment which entitle them to greater credit than they get. For that reason I see no sense in the frequent opposition to their representation at conventions. I could see more reason if a convention would embrace an entire afternoon in an inspection and explanation of the dental goods.—*Editorial, Dominion Dental Journal.*

Treatment of Lead.—Sulphur added to molten lead will cause it to be clean and pliable when cooled.—*P. A. Mariotte, Pac. Den. Gazette.*

